# Objective nutritional indices as an independent predictor of functional outcome after endovascular therapy for acute ischemic stroke: a cohort study in a Chinese population

**DOI:** 10.3389/fnut.2025.1504208

**Published:** 2025-06-18

**Authors:** Weiwei Gao, Lingfeng Yu, Yifen Zhang, Shouyue Jin, Zhongjie Chen, Xingyu Chen, Lijuan Cai, Renjing Zhu

**Affiliations:** ^1^Department of Neurology, Zhongshan Hospital of Xiamen University, School of Medicine, Xiamen University, Xiamen, China; ^2^Xiamen Clinical Research Center for Cerebrovascular Diseases, Xiamen, China; ^3^Xiamen Quality Control Center for Stroke, Xiamen, China; ^4^School of Medicine, Xiamen University, Xiamen, China; ^5^The School of Clinical Medicine, Fujian Medical University, Fuzhou, Fujian, China

**Keywords:** acute ischemic stroke, large vessel occlusion, endovascular therapy, nutritional status, Prognostic Nutritional Index, Controlling Nutritional Status, hemoglobin-albumin-lymphocyte-platelet score

## Abstract

**Objective:**

To investigate the associations between three nutritional indices—Prognostic Nutritional Index (PNI), Controlling Nutritional Status (CONUT) score, and Hemoglobin-Albumin-Lymphocyte-Platelet (HALP) score—and 90-day functional outcomes in patients with large vessel occlusion acute ischemic stroke (LVO-AIS) who underwent endovascular therapy (EVT).

**Methods:**

In this retrospective cohort study, we consecutively enrolled 409 LVO-AIS patients who received EVT at a comprehensive stroke center between January 2019 and December 2024. The primary endpoint was poor functional outcome, defined as a modified Rankin Scale (mRS) score of 3–6 at 90 days. Associations between nutritional indices and functional outcomes were analyzed using multivariable logistic regression models with stepwise adjustment. Restricted cubic spline (RCS) analysis was performed to explore potential non-linear relationships. Subgroup analyses with interaction tests assessed the consistency of these associations across demographic and clinical subgroups.

**Results:**

At 90-day follow-up, 261 patients (63.8%) had poor functional outcomes. The prevalence of malnutrition risk varied substantially between nutritional indices: PNI identified 15.9% of patients at risk, whereas the CONUT scoring system classified 81.9% of patients as having some degree of malnutrition risk. After comprehensive adjustment for confounders, each one-unit increase in PNI was associated with a 6% reduction in the risk of poor outcomes (adjusted OR = 0.94, 95% CI: 0.89–0.99, *p* = 0.026), while each one-unit increase in HALP score was associated with a 3% reduction (adjusted OR = 0.97, 95% CI: 0.96–0.99, *p* = 0.001). RCS analysis revealed a significant non-linear relationship between HALP score and functional outcomes (*P*-non-linear = 0.021), characterized by a steep risk reduction as scores increased at lower values, followed by a plateau effect. Subgroup analyses demonstrated consistent associations between nutritional indices and outcomes across various demographic and clinical characteristics, with no significant interaction effects observed.

**Conclusion:**

Prognostic Nutritional Index and HALP scores serve as independent predictors of poor 90-day functional outcomes in LVO-AIS patients treated with EVT. The significant non-linear relationship observed between HALP score and functional outcomes suggests that interventions targeting patients with moderate to severe malnutrition risk may yield greater clinical benefits.

## 1 Introduction

Large vessel occlusion acute ischemic stroke (LVO-AIS) accounts for approximately 15–30% of all acute ischemic stroke (AIS) cases and represents a significant clinical challenge due to its rapid progression, severe neurological deficits, and poor prognosis (1–3). Endovascular therapy (EVT), by achieving rapid vessel recanalization and salvaging the ischemic penumbra, has significantly improved patient outcomes and has become the standard treatment strategy for LVO-AIS patients in the hyperacute phase (4). Nevertheless, a substantial proportion of patients still experience poor functional recovery despite successful reperfusion, suggesting the existence of other prognostic factors that warrant further investigation (5).

Growing evidence indicates that malnutrition may be a critical factor influencing the pathophysiological processes and recovery trajectory of AIS (6–20). Compared to patients with non-LVO AIS, those with LVO-AIS face a more pronounced risk of malnutrition due to larger infarct volumes, more severe neurological deficits, higher incidence of dysphagia, difficulties in implementing nutritional support, and increased risk of complications. The interaction between neurological injury and nutritional status creates a potential vicious cycle, wherein malnutrition exacerbates neurological damage, while neurological deficits further compromise nutritional intake (6, 7). Consequently, comprehensive nutritional assessment and early intervention may significantly improve quality of life and clinical outcomes in this high-risk population (7).

Several validated nutritional indices have demonstrated prognostic value in stroke populations. The Prognostic Nutritional Index (PNI) and Controlling Nutritional Status (CONUT) score, as objective and clinically accessible nutritional risk screening tools, have been confirmed to be closely associated with various adverse outcomes in AIS patients, including early neurological deterioration, stroke-associated pneumonia, functional prognosis, mortality risk, and long-term stroke recurrence risk (8–14). However, evidence regarding the prognostic value of these nutritional indices in LVO-AIS patients undergoing EVT remains limited (15, 16). Additionally, the Hemoglobin-Albumin-Lymphocyte-Platelet (HALP) score, a novel index that comprehensively reflects systemic inflammatory response and nutritional status, has recently garnered widespread attention in stroke research (17–20). Although preliminary studies have explored the associations between HALP score and various clinical outcomes in AIS, its prognostic predictive value in patients receiving EVT awaits systematic validation.

Based on this background, the present study aims to investigate the baseline malnutrition risk status in LVO-AIS patients receiving EVT and to evaluate the associations between the three nutritional indices and 90-day functional outcomes. We hypothesize that poor nutritional status is an independent predictor of poor functional outcomes after EVT treatment in LVO-AIS patients. By elucidating these associations, we hope to provide evidence-based guidance for early identification of high-risk patients and the development of individualized nutritional intervention strategies, ultimately optimizing the comprehensive management approach for LVO-AIS patients receiving EVT and improving their long-term clinical prognosis.

## 2 Materials and methods

### 2.1 Study design and population

We conducted a single-center retrospective cohort study using data from a comprehensive stroke center in China. We consecutively enrolled adult patients with LVO-AIS who underwent EVT between January 1, 2019, and December 31, 2024. Large vessel occlusion was confirmed in all patients prior to EVT using computed tomography angiography or magnetic resonance angiography, and further verified during the procedure by digital subtraction angiography (DSA). Eligible occlusion sites included the internal carotid artery, middle cerebral artery (M1 or M2 segment), vertebrobasilar artery, or posterior cerebral artery (P1 segment).

Exclusion criteria were: (1) intracranial hemorrhage on baseline head computed tomography or magnetic resonance imaging, as hemorrhage represents a contraindication to endovascular reperfusion therapy; (2) pre-stroke modified Rankin Scale (mRS) score >2, as significant pre-existing disability would confound the assessment of post-stroke functional outcomes; (3) severe systemic comorbidities, including renal failure, severe hepatic dysfunction, or active malignancy, which could significantly impact nutritional assessment and clinical outcomes; (4) incomplete important clinical data, including procedural parameters; or (5) loss to follow-up at 90 days. The patient screening process is detailed in Supplementary Figure 1. The study protocol was approved by the institutional ethics committee and conducted in accordance with the Declaration of Helsinki. The ethics committee waived the requirement for informed consent given the retrospective design and anonymization of patient data.

### 2.2 Data collection

We extracted baseline data from electronic medical records, including demographic characteristics (age and sex), cerebrovascular risk factors [smoking status, alcohol consumption, hypertension, diabetes mellitus, hyperlipidemia, atrial fibrillation, coronary artery disease, valvular heart disease, and history of previous stroke or transient ischemic attack (TIA)], and vital signs at admission (systolic and diastolic blood pressure). Stroke severity was assessed at admission using the National Institutes of Health Stroke Scale (NIHSS). Stroke etiology was classified according to the Trial of Org 10,172 in Acute Stroke Treatment (TOAST) criteria. Due to the limited number of patients with other determined or undetermined etiologies, these categories were combined for analysis.

Procedure-related parameters were documented in real-time by interventional neuroradiologists, including information on intravenous thrombolysis, key time metrics (onset-to-puncture time, onset-to-reperfusion time, and puncture-to-reperfusion time), and procedural details [number of mechanical thrombectomy attempts, device strategy (stent retriever, aspiration catheter, or combined approach), and balloon angioplasty]. Reperfusion status was evaluated based on final DSA results using the modified Thrombolysis in Cerebral Infarction coring system, with mTICI grades 2b-3 defined as successful reperfusion.

Laboratory specimens were collected from all patients in a fasting state within 24 h of admission. All tests were performed in the central clinical laboratory using standardized methods and automated analyzers that underwent regular quality control and calibration. Laboratory parameters included hematological indices (white blood cell, neutrophils, lymphocytes, monocytes, Red blood cells, hemoglobin concentration, and platelets) and biochemical markers (total protein, serum albumin, triglycerides, total cholesterol, high-density lipoprotein cholesterol, low-density lipoprotein cholesterol, aspartate aminotransferase [AST], alanine aminotransferase, serum creatinine, and uric acid). Patients with completely missing hematological or biochemical data were excluded. For patients with only partial missing indicators and an overall missing rate of less than 10%, we employed a random forest multiple imputation algorithm to preserve sample size and minimize selection bias.

### 2.3 Nutritional indices

We assessed the nutritional status of all enrolled patients using three objective nutritional indices: PNI, CONUT score, and HALP score. The PNI was calculated using the formula: PNI = serum albumin (g/L) + 5 × lymphocytes (× 10^9^/L). Based on previous literature (21), we stratified patients into three groups according to PNI scores: normal nutritional status (>38 points), moderate malnutrition risk (35–38 points), and severe malnutrition risk (<35 points).

The CONUT scoring criteria were as follows (11): (1) serum albumin: >35 g/L (0 points), 30–34.99 g/L (2 points), 25–29.99 g/L (4 points), <25 g/L (6 points); (2) total cholesterol: ≥4.66 mmol/L (0 points), 3.63–4.65 mmol/L (1 point), 2.59–3.62 mmol/L (2 points), <2.59 mmol/L (3 points); (3) lymphocyte count: ≥1.6 × 10^9^/L (0 points), 1.2–1.59 × 10^9^/L (1 point), 0.8–1.19 × 10^9^/L (2 points), <0.8 × 10^9^/L (3 points). The total CONUT score was the sum of these three parameters (range 0–12 points). Based on the total score, we categorized patients into three groups: normal nutritional status (0–1 points), mild malnutrition risk (2–4 points), and moderate-to-severe malnutrition risk (5–12 points).

The HALP score was calculated using the formula: HALP = [hemoglobin (g/L) × serum albumin (g/L) × lymphocytes (10^9^/L)]/platelet (10^9^/L).

### 2.4 Outcome measures

The primary endpoint of this study was functional outcome at 90 days after EVT (22), assessed using the mRS. An mRS score of 0–2 at 90 days (indicating functional independence in daily activities) was defined as a good outcome, while an mRS score of 3–6 (indicating varying degrees of functional dependence or death) was defined as a poor outcome.

All follow-up data were collected and managed through the National Cerebrovascular Disease Big Data Platform (Stroke Center Construction Information Management System). The 90-day mRS assessments were conducted via structured telephone interviews by follow-up personnel who had received training and certification in the mRS assessment system, with results promptly recorded in the system. To ensure data quality and reliability, two professionally trained neurologists independently collected and recorded all data according to standardized procedures, followed by cross-verification by other research personnel.

### 2.5 Statistical analysis

All statistical analyses were performed using R software (version 4.2.2). The normality of continuous variables was assessed using the Shapiro–Wilk test. Normally distributed continuous variables were presented as mean ± standard deviation (SD) and compared between groups using independent sample *t*-tests; non-normally distributed continuous variables were expressed as median and interquartile range (IQR) and analyzed using the Mann–Whitney *U* test. Categorical variables were presented as frequencies and percentages [*n* (%)] and compared using the Pearson *χ*^2^ test or Fisher’s exact test as appropriate.

To evaluate the predictive value of nutritional indices for clinical outcomes, we constructed receiver operating characteristic (ROC) curves and calculated the area under the curve (AUC), sensitivity, and specificity. Optimal cutoff values were determined using the Youden index. Differences in AUCs between different nutritional assessment tools were compared using the DeLong test.

Multicollinearity among variables was systematically assessed before multivariable analysis. Spearman rank correlation analysis was performed on covariates with statistical significance (*p* < 0.05) in univariate analysis, and variance inflation factors (VIFs) were calculated. Correlation strength was classified according to the following criteria: |*r*| ≤ 0.3 (negligible linear correlation), 0.3 < |*r*| ≤ 0.5 (weak linear correlation), 0.5 < |*r*| ≤ 0.8 (moderate linear correlation), and |*r*| > 0.8 (strong linear correlation). Multicollinearity was evaluated using the following VIF thresholds: VIF < 5 (low collinearity), 5 ≤ VIF < 10 (moderate collinearity), and VIF ≥ 10 (severe collinearity). Given the significant correlation between lymphocyte count and the PNI, CONUT, and HALP scores (*r* > 0.5), and the fact that it is a component of these scores, we excluded it from the multivariable analysis. Similarly, because white blood cell count was highly correlated with neutrophil count (*r* = 0.98, VIF ≥ 10), only white blood cell count was retained in the multivariable models (Supplementary Table 1).

To investigate the independent associations between the three nutritional indices and poor functional outcomes, we constructed three progressively adjusted multivariable logistic regression models: Model 1 (unadjusted); Model 2 (adjusted for age, current smoker, hypertension, diabetes mellitus, atrial fibrillation, and baseline NIHSS score); and Model 3 (further adjusted for number of thrombectomy attempts, puncture-to-reperfusion time, white blood cell, Red blood cells, platelet, and AST). The performance of the nutritional score prediction models was comprehensively evaluated using multiple indicators (Supplementary Table 2): goodness-of-fit indicators (AIC, AICc, BIC, and their corresponding weights); discriminative ability (Tjur’s *R*^2^); predictive accuracy indicators (RMSE, Sigma, and Log_loss); calibration indicators (Score_log and Score_spherical); and classification performance (PCP).

To explore potential non-linear relationships between nutritional indices and outcomes, we employed restricted cubic spline (RCS) regression models, with knot placement determined based on the principle of AIC minimization (Supplementary Table 3). Given that the CONUT score is a discrete integer scoring system (range 0–12 points) with extremely sparse sample distribution in the high-score region (8–12 points), resulting in highly uneven data distribution, it was not suitable for non-linear relationship analysis using the RCS model, which relies on assumptions of data continuity and uniform distribution. Based on previous evidence and biological plausibility, we conducted subgroup analyses according to sex, age (<60 years vs. ≥60 years), and major vascular risk factors (hypertension, diabetes, hyperlipidemia, atrial fibrillation, history of previous stroke or TIA, coronary artery disease, and valvular heart disease), and assessed between-subgroup effect heterogeneity through interaction term testing. All statistical tests were two-sided, with *p* < 0.05 considered statistically significant.

## 3 Results

### 3.1 Baseline characteristics and laboratory parameters

Among the 409 LVO-AIS patients who underwent EVT (Table 1), the median age was 67 years (IQR, 57–76), with a predominance of males (*n* = 273, 66.8%). At 90-day follow-up, 148 patients (36.2%) achieved good functional outcomes (mRS 0–2), while 261 patients (63.8%) had poor outcomes (mRS 3–6). Compared with patients with good outcomes, those with poor outcomes were significantly older (median: 69 vs. 63 years, *p* < 0.001) and presented with more severe neurological deficits at admission (median NIHSS score: 17 vs. 12, *p* < 0.001). The poor outcome group had a higher prevalence of vascular risk factors, including hypertension (*p* = 0.037), diabetes mellitus (*p* = 0.048), and atrial fibrillation (*p* = 0.004). Conversely, the proportion of smokers was significantly higher in the good outcome group (*p* = 0.022). Regarding procedural characteristics, patients with good outcomes had significantly shorter puncture-to-reperfusion times (median: 70 vs. 81 min, *p* < 0.001) and fewer thrombectomy attempts (median: 1 vs. 2 attempts, *p* = 0.020).

**Table 1:** Baseline demographic, clinical, and procedural characteristics stratified by 90-day functional outcome after endovascular therapy for large vessel occlusion ischemic stroke.

Characteristics	Overall (*n* = 409)	Good outcome (*n* = 148)	Poor outcome (*n* = 261)	*p*-value
Age, years	67 (57, 76)	63 (53, 72)	69 (58, 77)	<0.001
Sex, male	273 (66.75)	104 (70.27)	169 (64.75)	0.255
Current smoker	150 (36.67)	65 (43.92)	85 (32.57)	0.022
Alcohol consumption	98 (23.96)	37 (25.00)	61 (23.37)	0.711
Medical history				
Hypertension	275 (67.24)	90 (60.81)	185 (70.88)	0.037
Diabetes mellitus	118 (28.85)	34 (22.97)	84 (32.18)	0.048
Hyperlipidemia	99 (24.21)	42 (28.38)	57 (21.84)	0.138
Atrial fibrillation	165 (40.34)	46 (31.08)	119 (45.59)	0.004
Previous stroke or TIA	62 (15.16)	20 (13.51)	42 (16.09)	0.485
Coronary artery disease	48 (11.74)	16 (10.81)	32 (12.26)	0.662
Valvular heart disease	56 (13.69)	16 (10.81)	40 (15.33)	0.202
Clinical presentation				
SBP, mmHg	148 (133, 164)	147 (133, 161)	150 (133, 167)	0.211
DBP, mmHg	87 (77, 97)	86 (75, 97)	88 (78, 98)	0.232
Baseline NIHSS score	15 (11, 19)	12 (8, 15)	17 (13, 20)	<0.001
Stroke etiology				0.103
Large-artery atherosclerosis	212 (51.83)	85 (57.43)	127 (48.66)	
Cardioembolism	177 (43.28)	54 (36.49)	123 (47.13)	
Other	20 (4.89)	9 (6.08)	11 (4.21)	
Occlusion location				
Anterior circulation	345 (84.35)	223 (85.44)	122 (82.43)	0.421
Posterior circulation	70 (17.11)	45 (17.24)	25 (16.89)	0.928
Procedural Characteristics				
Intravenous thrombolysis	168 (41.08)	65 (43.92)	103 (39.46)	0.379
OPT, min	369 (259, 560)	342 (230, 560)	378 (268, 559)	0.351
PRT, min	80 (53, 105)	70 (41, 99)	81 (60, 120)	<0.001
ORT, min	463 (345, 666)	445 (315, 677)	477 (358, 655)	0.164
NOTA	2.00 (1.00, 2.00)	1.00 (1.00, 2.00)	2.00 (1.00, 3.00)	0.020
Successful reperfusion	310 (75.79)	120 (81.08)	190 (72.80)	0.060
Endovascular technique				0.199
Stent retriever only	97 (23.72)	35 (23.65)	62 (23.75)	
Aspiration only	25 (6.11)	11 (7.43)	14 (5.36)	
Combined approach	256 (62.59)	96 (64.86)	160 (61.30)	
Balloon angioplasty	88 (21.52)	33 (22.30)	55 (21.07)	0.772

Values are presented as median (interquartile range) or number (percentage). Good outcome was defined as a mRS score of 0–2 at 90 days. Successful reperfusion was defined as a modified Thrombolysis in Cerebral Infarction (mTICI) score of 2b-3. mRS, modified Rankin Scale; TIA, transient ischemic attack; SBP, systolic blood pressure; DBP, diastolic blood pressure; NIHSS, National Institutes of Health Stroke Scale; OPT, onset-to-puncture time; PRT, puncture-to-reperfusion time; ORT, onset-to-reperfusion time; NOTA, number of thrombectomy attempts.

Table 2 summarizes the laboratory parameters. Compared with patients with good outcomes, those with poor outcomes exhibited a more pronounced inflammatory response, characterized by significantly elevated white blood cell counts (median: 12.0 vs. 10.6 × 10^9^/L, *p* < 0.001) and neutrophil counts (median: 10.4 vs. 8.7 × 10^9^/L, *p* < 0.001), alongside significantly reduced lymphocyte counts (median: 0.92 vs. 1.24 × 10^9^/L, *p* < 0.001). Additionally, patients with poor outcomes had significantly lower red blood cell counts (median: 4.13 vs. 4.29 × 10^12^/L, *p* = 0.040) and platelet counts (median: 199 vs. 213 × 10^9^/L, *p* = 0.018). Among biochemical indicators, only AST levels differed significantly between the groups, with higher levels observed in the poor outcome group (median: 26 vs. 23 U/L, *p* = 0.003).

**Table 2:** Laboratory parameters and nutritional assessment scores according to 90-day functional outcome after endovascular therapy for large vessel occlusion stroke.

Parameter	Overall (*n* = 409)	Good outcome (*n* = 148)	Poor outcome (*n* = 261)	*p*-value
Hematologic parameters				
White blood cell, ×10^9^/L	11.6 (9.6, 13.9)	10.6 (8.7, 12.9)	12.0 (10.2, 14.6)	<0.001
Neutrophils, ×10^9^/L	9.9 (7.9, 11.9)	8.7 (6.9, 10.8)	10.4 (8.5, 12.8)	<0.001
Lymphocytes, ×10^9^/L	1.04 (0.73, 1.38)	1.24 (0.92, 1.63)	0.92 (0.68, 1.21)	<0.001
Monocytes, ×10^9^/L	0.59 (0.45, 0.75)	0.59 (0.46, 0.73)	0.59 (0.43, 0.77)	0.796
Red blood cells, ×10^12^/L	4.19 (3.73, 4.63)	4.29 (3.92, 4.60)	4.13 (3.65, 4.65)	0.040
Hemoglobin, g/L	128 (115, 141)	132 (118, 141)	126 (112, 142)	0.100
Platelet, ×10^9^/L	203 (170, 239)	213 (180, 247)	199 (163, 234)	0.018
Biochemical parameters				
Total Protein, g/L	65 (62, 70)	65 (61, 69)	65 (62, 70)	0.245
Albumin, g/L	37.2 (34.6, 39.7)	37.1 (35.2, 39.5)	37.3 (34.3, 39.7)	0.833
ALT, U/L	16 (12, 24)	17 (12, 26)	16 (12, 23)	0.154
AST, U/L	25 (19, 32)	23 (18, 28)	26 (20, 36)	0.003
Triglycerides, mmol/L	1.08 (0.80, 1.48)	1.12 (0.82, 1.43)	1.07 (0.77, 1.48)	0.491
Total cholesterol, mmol/L	4.35 (3.62, 5.03)	4.34 (3.74, 4.94)	4.35 (3.52, 5.12)	0.827
HDL cholesterol, mmol/L	1.16 (0.99, 1.34)	1.15 (1.00, 1.30)	1.16 (0.99, 1.35)	0.451
LDL cholesterol, mmol/L	2.87 (2.24, 3.39)	2.87 (2.37, 3.42)	2.87 (2.15, 3.33)	0.302
Creatinine, μmol/L	72 (59, 88)	72 (58, 83)	73 (59, 90)	0.098
Uric acid, μmol/L	324 (266, 409)	316 (265, 391)	329 (269, 417)	0.156
Nutritional assessment scores				
Prognostic Nutritional Index	42.5 (39.7, 45.4)	43.5 (41.1, 46.3)	41.9 (39.2, 44.5)	<0.001
Nutritional status by PNI				0.025
Normal (>38)	344 (84.11)	133 (89.86)	211 (80.84)	
Moderate (35–38)	37 (9.05)	11 (7.43)	26 (9.96)	
Severe (<35)	28 (6.85)	4 (2.70)	24 (9.20)	
COUNT score	3.00 (2.00, 5.00)	2.50 (1.00, 4.00)	4.00 (2.00, 5.00)	<0.001
Nutritional status by CONUT				<0.001
Normal (0–1)	74 (18.09)	44 (29.73)	30 (11.49)	
Mild (2–4)	230 (56.23)	74 (50.00)	156 (59.77)	
Moderate to severe (5–12)	105 (25.67)	30 (20.27)	75 (28.74)	
HALP score	25 (16, 33)	28 (20, 39)	21 (15, 30)	<0.001
HALP quartiles				<0.001
Quartile 1 [3.69,15.9]	102 (24.94)	23 (15.54)	79 (30.27)	
Quartile 2 [15.9,24.6]	102 (24.94)	29 (19.59)	73 (27.97)	
Quartile 3 [24.6,32.8]	102 (24.94)	39 (26.35)	63 (24.14)	
Quartile 4 [32.8,138]	103 (25.18)	57 (38.51)	46 (17.62)	

Values are presented as median (interquartile range) or number (percentage). Good outcome was defined as a mRS score of 0–2 at 90 days. mRS, modified Rankin Scale; ALT, alanine aminotransferase; AST, aspartate aminotransferase; HDL, high-density lipoprotein; LDL, low-density lipoprotein; HALP, hemoglobin, albumin, lymphocyte, and platelet; PNI, Prognostic Nutritional Index; CONUT, Controlling Nutritional Status.

### 3.2 Nutritional status assessment

The proportion of patients identified as having malnutrition risk varied substantially between different nutritional indices (Table 2). Based on PNI thresholds, 15.9% of the entire cohort was at risk of malnutrition, with 9.1% classified as having moderate malnutrition risk (PNI 35–38) and 6.8% as having severe malnutrition risk (PNI < 35). In contrast, when using the CONUT scoring system, up to 81.9% of patients exhibited varying degrees of malnutrition risk, including 56.2% with mild malnutrition risk (CONUT 2–4) and 25.7% with moderate-to-severe malnutrition risk (CONUT 5–12).

**Figure 1:**
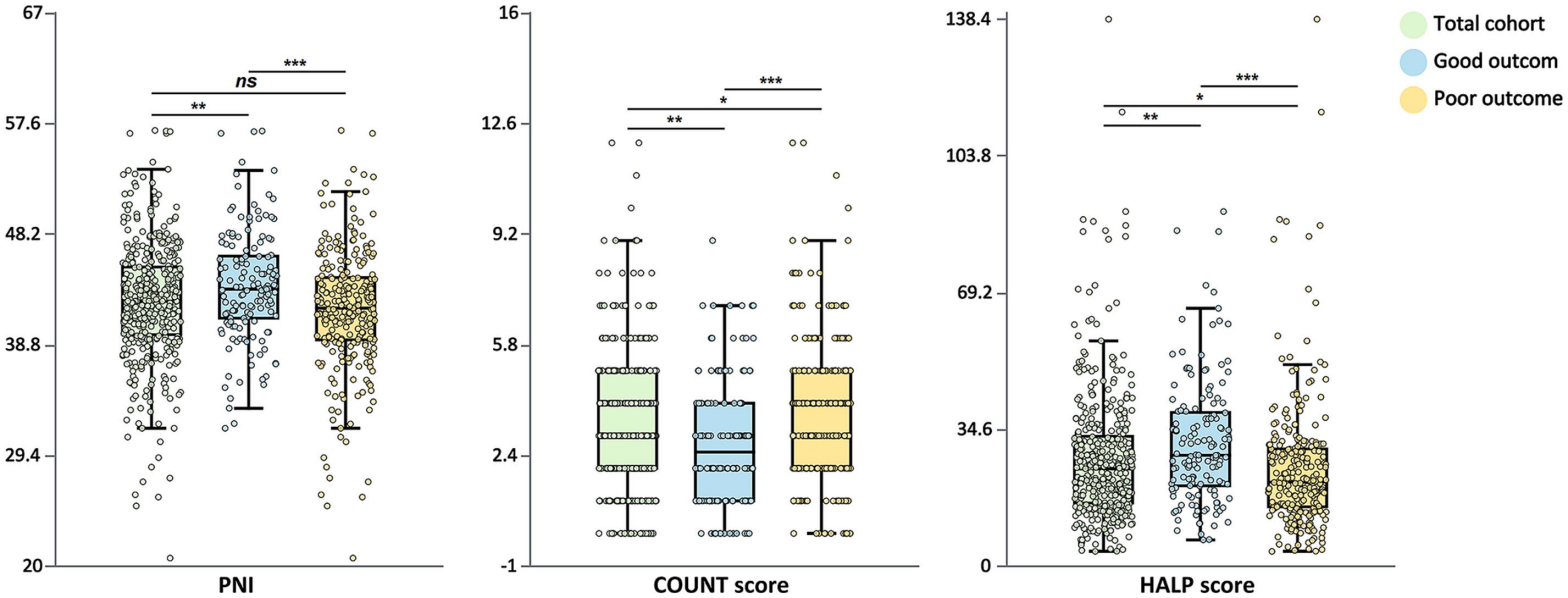
Distribution of nutritional indices by functional outcome. Box plots showing the distribution of PNI, CONUT score, and HALP score stratified by functional outcome at 90 days. The total cohort (green), good outcome group (mRS 0–2, blue), and poor outcome group (mRS 3–6, yellow) are displayed. The central line in each box represents the median, the box limits indicate the interquartile range, and the whiskers extend to 1.5 times the interquartile range. Individual data points are overlaid. Statistical significance: *p < 0.05, **p < 0.01, ***p < 0.001, ns, not significant. PNI, Prognostic Nutritional Index; CONUT, Controlling Nutritional Status; HALP, hemoglobin-albumin-lymphocyte-platelet; mRS, modified Rankin Scale.

Patients with poor functional outcomes had significantly lower PNI values compared to those with good outcomes (median: 41.9 vs. 43.5, *p* < 0.001; Figure 1). Nutritional status classification based on PNI also differed significantly between the groups (*p* = 0.025), with normal nutritional status being more prevalent in the good outcome group (89.9% vs. 80.8%), while severe malnutrition risk was more common in the poor outcome group (9.2% vs. 2.7%).

Similarly, CONUT scores were significantly higher in patients with poor outcomes compared to those with good outcomes (median: 4.00 vs. 2.50, *p* < 0.001). The distribution of CONUT score categories differed significantly between groups (*p* < 0.001), with normal nutritional status being more common in the good outcome group (29.7% vs. 11.5%), while moderate-to-severe malnutrition risk was more prevalent in the poor outcome group (28.7% vs. 20.3%).

HALP scores were significantly lower in patients with poor outcomes (median: 21 vs. 28, *p* < 0.001). Quartile analysis of HALP scores revealed that patients with good outcomes constituted a larger proportion in the highest quartile (38.5% vs. 17.6%), whereas patients with poor outcomes were more concentrated in the lowest quartile (30.3% vs. 15.5%) (*p* < 0.001).

**Figure 2:**
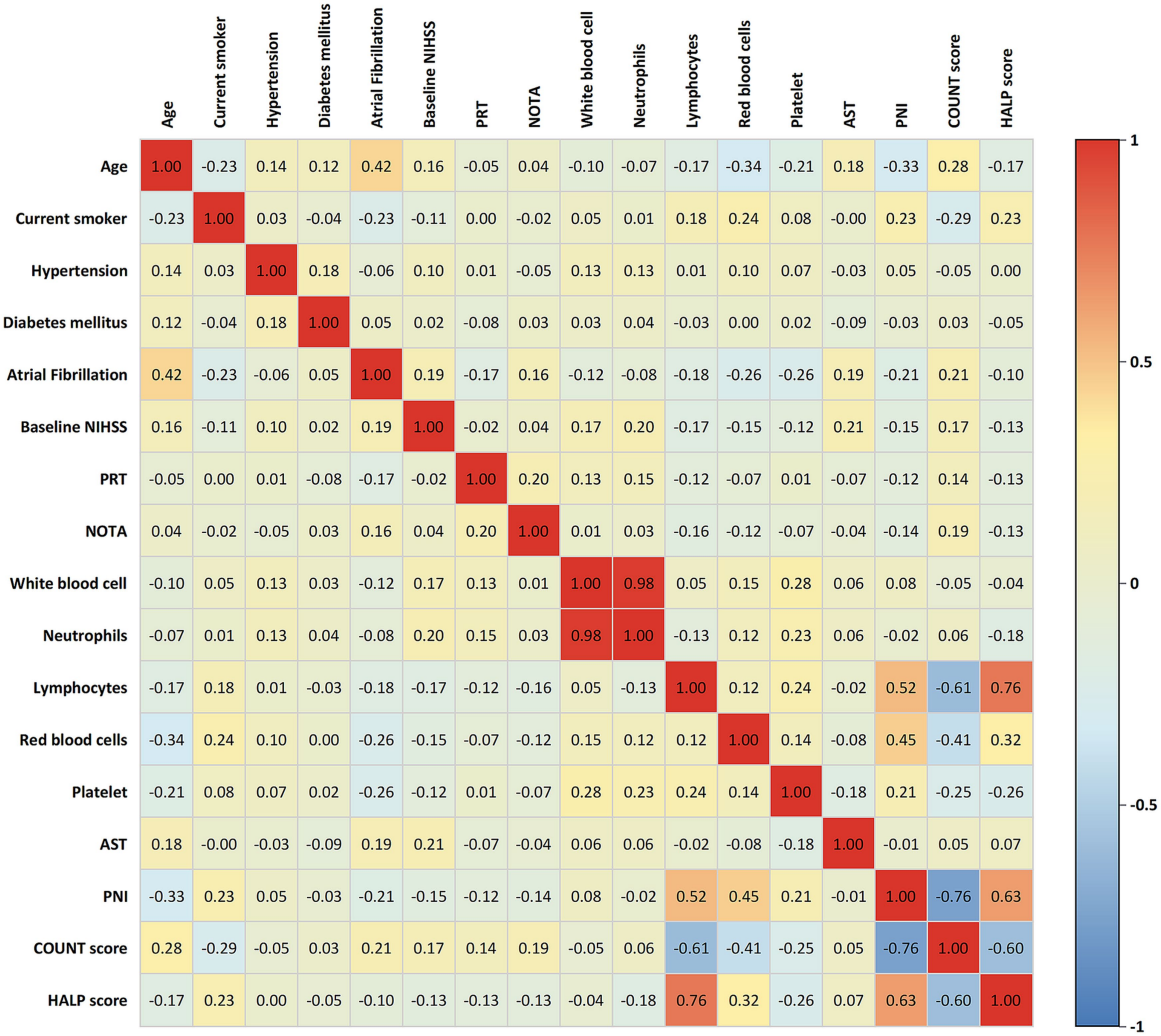
Correlation matrix of clinical parameters and nutritional indices in LVO-AIS patients. Color intensity and numerical values represent correlation strength, with red indicating positive correlation and blue indicating negative correlation. PNI, Prognostic Nutritional Index; CONUT, Controlling Nutritional Status; HALP, hemoglobin-albumin-lymphocyte-platelet; NIHSS, National Institutes of Health Stroke Scale; PRT, puncture-to-reperfusion time; NOTA, number of thrombectomy attempts; AST, aspartate aminotransferase.

Spearman correlation analysis (Figure 2) demonstrated strong associations among the three nutritional assessment indices. PNI showed a strong negative correlation with CONUT score (*r* = −0.76) and a strong positive correlation with HALP score (*r* = 0.63). Likewise, CONUT score exhibited a strong negative correlation with HALP score (*r* = −0.60). These correlation patterns confirmed the internal consistency among these nutritional assessment tools.

### 3.3 Predictive value of nutritional indices for functional outcomes

**Figure 3:**
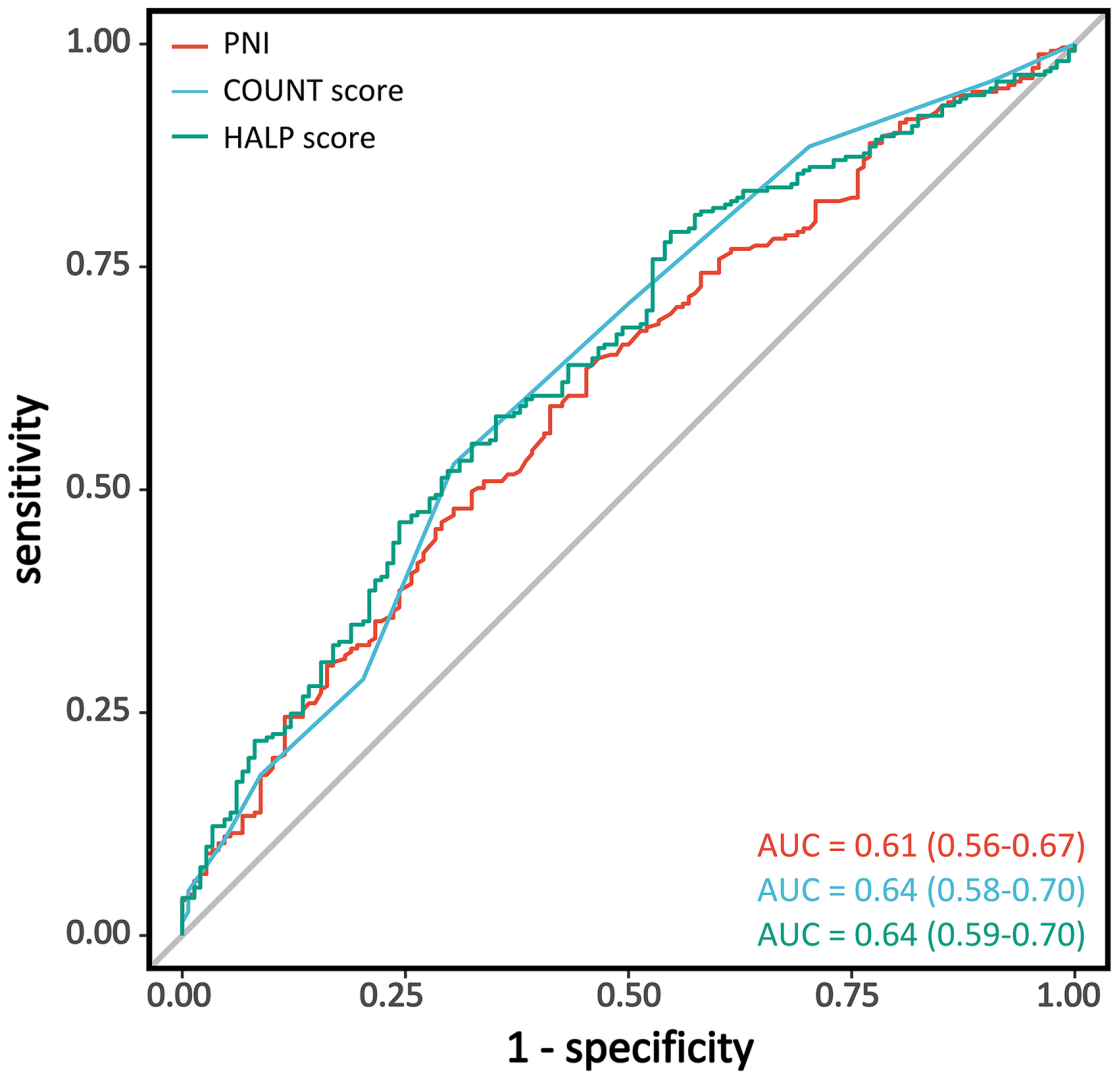
Receiver operating characteristic curves for nutritional indices in predicting poor functional outcomes. Receiver operating characteristic (ROC) curves showing the discriminative ability of PNI (red), CONUT score (light blue), and HALP score (green) for predicting poor functional outcomes (mRS 3–6) at 90 days after endovascular therapy. PNI, Prognostic Nutritional Index; CONUT, Controlling Nutritional Status; HALP, hemoglobin-albumin-lymphocyte-platelet; AUC, area under the curve; mRS, modified Rankin Scale.

We evaluated the discriminative ability of the three nutritional assessment tools for poor functional outcomes after EVT using ROC curve analysis (Table 3 and Figure 3). All three indices demonstrated moderate predictive capability, with CONUT score showing the highest diagnostic performance (AUC = 0.64; 95% CI: 0.58–0.70), followed by HALP score with an identical AUC of 0.64 (95% CI: 0.59–0.70). PNI exhibited slightly lower predictive ability (AUC = 0.61; 95% CI: 0.56–0.67). Pairwise comparisons using the DeLong test indicated no statistically significant differences in discriminative performance among the three nutritional indices (all *p* > 0.05, Supplementary Figure 2).

**Table 3:** Diagnostic performance of nutritional assessment scores in predicting poor outcome after endovascular therapy for large vessel occlusion ischemic stroke.

Nutritional score	AUC (95% CI)	Accuracy (95% CI)	Sensitivity (95% CI)	Specificity (95% CI)	Cut-off calue
PNI	0.61 (0.56–0.67)	0.40 (0.35–0.45)	0.45 (0.37–0.53)	0.36 (0.31–0.42)	43.245
COUNT score	0.64 (0.58–0.70)	0.59 (0.54–0.64)	0.70 (0.62–0.77)	0.53 (0.47–0.59)	3.5
HALP score	0.64 (0.59–0.70)	0.33 (0.29–0.38)	0.55 (0.47–0.63)	0.21 (0.16–0.26)	30.947

Poor functional outcome was defined as a mRS score of 3–6 at 90 days post-procedure. Optimal cut-off values were determined using Youden’s index to maximize the sum of sensitivity and specificity. AUC, area under the receiver operating characteristic curve; CI, confidence interval; PNI, Prognostic Nutritional Index; CONUT, Controlling Nutritional Status; HALP, hemoglobin, albumin, lymphocyte, and platelet.

The optimal cutoff value for each nutritional index was determined using the Youden index. For CONUT score, a threshold of 3.5 provided the highest accuracy (0.59), with a sensitivity of 0.70 and specificity of 0.53. For PNI, the optimal cutoff value was 43.245, with an accuracy of 0.40, sensitivity of 0.45, and specificity of 0.36. The optimal threshold for HALP score was 30.947, with an accuracy of 0.33, sensitivity of 0.55, and specificity of 0.21. Among the three nutritional assessment tools, CONUT score demonstrated the best overall predictive performance, with notably higher sensitivity and accuracy compared to PNI and HALP scores.

### 3.4 Multivariable analysis of nutritional indices for functional outcomes

To further investigate the relationship between nutritional assessment indices and 90-day functional outcomes, we constructed three progressively adjusted logistic regression models (Table 4). When analyzed as continuous variables, all three nutritional indices were significantly associated with poor functional outcomes in both the unadjusted model (Model 1) and the partially adjusted model (Model 2, which controlled for age, current smoker, hypertension, diabetes mellitus, atrial fibrillation, and baseline NIHSS score) (all *p* < 0.05). In the fully adjusted model (Model 3, which further adjusted for number of thrombectomy attempts, puncture-to-reperfusion time, white blood cell, red blood cells, platelet, and AST), each one-unit increase in PNI was associated with a 6% reduction in the risk of poor outcomes (OR = 0.94, 95% CI: 0.89–0.99, *p* = 0.026), while each one-unit increase in HALP score was associated with a 3% reduction in risk (OR = 0.97, 95% CI: 0.96–0.99, *p* = 0.001). In contrast, the association between CONUT score and poor outcomes was attenuated and no longer statistically significant after comprehensive adjustment (OR = 1.18, 95% CI: 0.91–1.53, *p* = 0.217).

**Table 4:** Multivariable logistic regression models for nutritional assessment scores and poor functional outcome after endovascular therapy in patients with large vessel occlusion stroke.

Nutritional parameter	Model 1		Model 2		Model 3	
	OR (95% CI)	*p*-value	OR (95% CI)	*p*-value	OR (95% CI)	*p*-value
PNI (per unit increase)	0.92 (0.88–0.96)	<0.001	0.94 (0.90–0.99)	0.013	0.94 (0.89–0.99)	0.026
COUNT score (per unit increase)	1.43 (1.14–1.79)	0.002	1.25 (0.96–1.61)	0.097	1.18 (0.91–1.53)	0.217
HALP score (per unit increase)	0.98 (0.97–0.99)	<0.001	0.98 (0.97–0.99)	0.003	0.97 (0.96–0.99)	0.001
Nutritional status by PNI						
Normal (>38)	reference		reference		reference	
Moderate (35–38)	1.49 (0.71–3.12)	0.289	1.11 (0.49–2.49)	0.800	1.02 (0.43–2.43)	0.970
Severe (<35)	3.78 (1.28–11.14)	0.016	3.41 (1.10–10.60)	0.034	3.19 (0.94–10.85)	0.063
*P* for trend		0.009		0.046		0.103
Nutritional status by CONUT						
Normal (0–1)	reference		reference		reference	
Mild (2–4)	3.09 (1.80–5.31)	<0.001	2.59 (1.44–4.66)	0.001	2.48 (1.33–4.62)	0.004
Moderate to severe (5–12)	3.67 (1.96–6.87)	<0.001	2.24 (1.10–4.56)	0.026	1.87 (0.85–4.13)	0.122
*P* for trend		<0.001		0.035		0.116
HALP score quartiles						
Quartile 1 [3.69,15.9]	reference		reference		reference	
Quartile 2 [15.9,24.6]	0.73 (0.39–1.38)	0.336	0.8 (0.41–1.57)	0.518	0.78 (0.37–1.63)	0.506
Quartile 3 [24.6,32.8]	0.47 (0.25–0.87)	0.016	0.57 (0.30–1.11)	0.098	0.46 (0.22–0.97)	0.043
Quartile 4 [32.8,138]	0.23 (0.13–0.43)	<0.001	0.29 (0.15–0.56)	<0.001	0.2 (0.09–0.44)	<0.001
*P* for trend		<0.001		<0.001		<0.001

Values are expressed as odds ratios (ORs) with 95% confidence intervals (CIs) for poor functional outcome (mRS score 3–6 at 90 days). Model 1: Unadjusted. Model 2: Adjusted for demographic and clinical factors (age, Current smoker, hypertension, diabetes mellitus, atrial fibrillation, and baseline NIHSS score). Model 3: Additionally adjusted for procedural parameters and laboratory indices (number of thrombectomy attempts, puncture-to-reperfusion time, leukocytes, erythrocytes, platelet, and aspartate aminotransferase). PNI, Prognostic Nutritional Index; CONUT, Controlling Nutritional Status; HALP, hemoglobin, albumin, lymphocyte, and platelet; NIHSS, National Institutes of Health Stroke Scale.

Patients classified as having severe malnutrition risk according to PNI criteria (PNI < 35) exhibited significantly higher risk of poor functional outcomes compared to those with normal nutritional status (PNI > 38) in the unadjusted model (OR = 3.78, 95% CI: 1.28–11.14, *p* = 0.016), with a significant trend across categories (*P* for trend = 0.009). This association persisted in Model 2 (OR = 3.41, 95% CI: 1.10–10.60, *p* = 0.034) but was attenuated to borderline significance in the fully adjusted model (OR = 3.19, 95% CI: 0.94–10.85, *p* = 0.063; *P* for trend = 0.103).

For CONUT score categories, both mild malnutrition risk (CONUT 2–4) and moderate-to-severe malnutrition risk (CONUT 5–12) were significantly associated with increased risk of poor outcomes in the unadjusted model (both *p* < 0.001), with a significant dose–response relationship across categories (*P* for trend<0.001). In the fully adjusted model, only mild malnutrition risk remained significantly associated with poor outcomes (OR = 2.48, 95% CI: 1.33–4.62, *p* = 0.004), while the association for moderate-to-severe malnutrition risk was attenuated (OR = 1.87, 95% CI: 0.85–4.13, *p* = 0.122).

When stratified by quartiles, patients in the highest HALP score quartile consistently demonstrated significantly lower risk of poor outcomes compared to those in the lowest quartile across all models, with this association strengthened after comprehensive adjustment (OR = 0.20, 95% CI: 0.09–0.44, *p* < 0.001). A robust dose–response relationship was observed across HALP score quartiles (*P* for trend<0.001 in all models), with the risk of poor outcomes progressively decreasing as HALP scores increased.

### 3.5 Restricted cubic spline analysis of PNI and HALP scores

**Figure 4:**
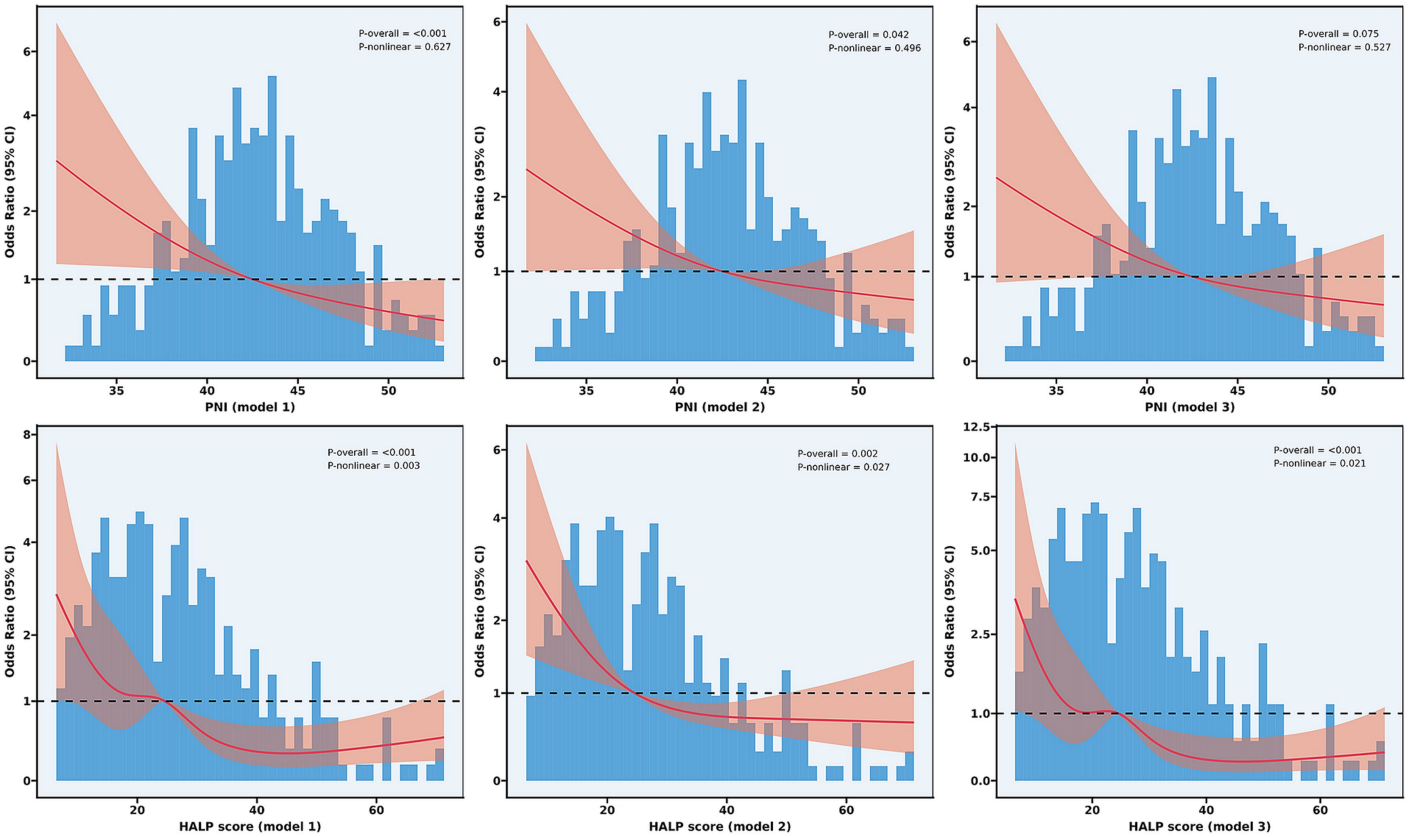
Non-linear relationship between nutritional indices and functional outcomes. Restricted cubic spline analyses showing the relationship between PNI (upper panels) and HALP score (lower panels) with odds ratios for poor functional outcomes at 90 days. Model 1 (unadjusted); Model 2 (adjusted for age, current smoker, hypertension, diabetes mellitus, atrial fibrillation, and baseline NIHSS score); and Model 3 (further adjusted for number of thrombectomy attempts, puncture-to-reperfusion time, white blood cell, red blood cells, platelet, and AST). PNI, Prognostic Nutritional Index; HALP, hemoglobin-albumin-lymphocyte-platelet.

To explore potential non-linear relationships between nutritional indices and functional outcomes, we conducted RCS analysis (Figure 4). For PNI, a significant overall association with poor functional outcomes was observed in the unadjusted model (*P* for overall<0.001), which remained significant after adjusting for demographic and clinical factors (*P* for overall = 0.042), but was attenuated to borderline significance in the fully adjusted model (*P* for overall = 0.075). The relationship between PNI and poor outcomes demonstrated predominantly linear characteristics, with non-linearity tests failing to reach statistical significance in all models (*P* for non-linearity: 0.627, 0.496, and 0.527, respectively).

In contrast, HALP score not only exhibited significant overall associations (*P* for overall<0.001 in all models) but also demonstrated significant non-linear relationships with poor functional outcomes (*P* for non-linearity: 0.003, 0.027, and 0.021 in Models 1, 2, and 3, respectively). The RCS analysis revealed a more complex relationship, characterized by a steep decline in the risk of poor outcomes as HALP scores increased from low to moderate values (approximately 15–40), followed by a plateau effect at higher HALP values.

### 3.6 Subgroup analyses

**Figure 5:**
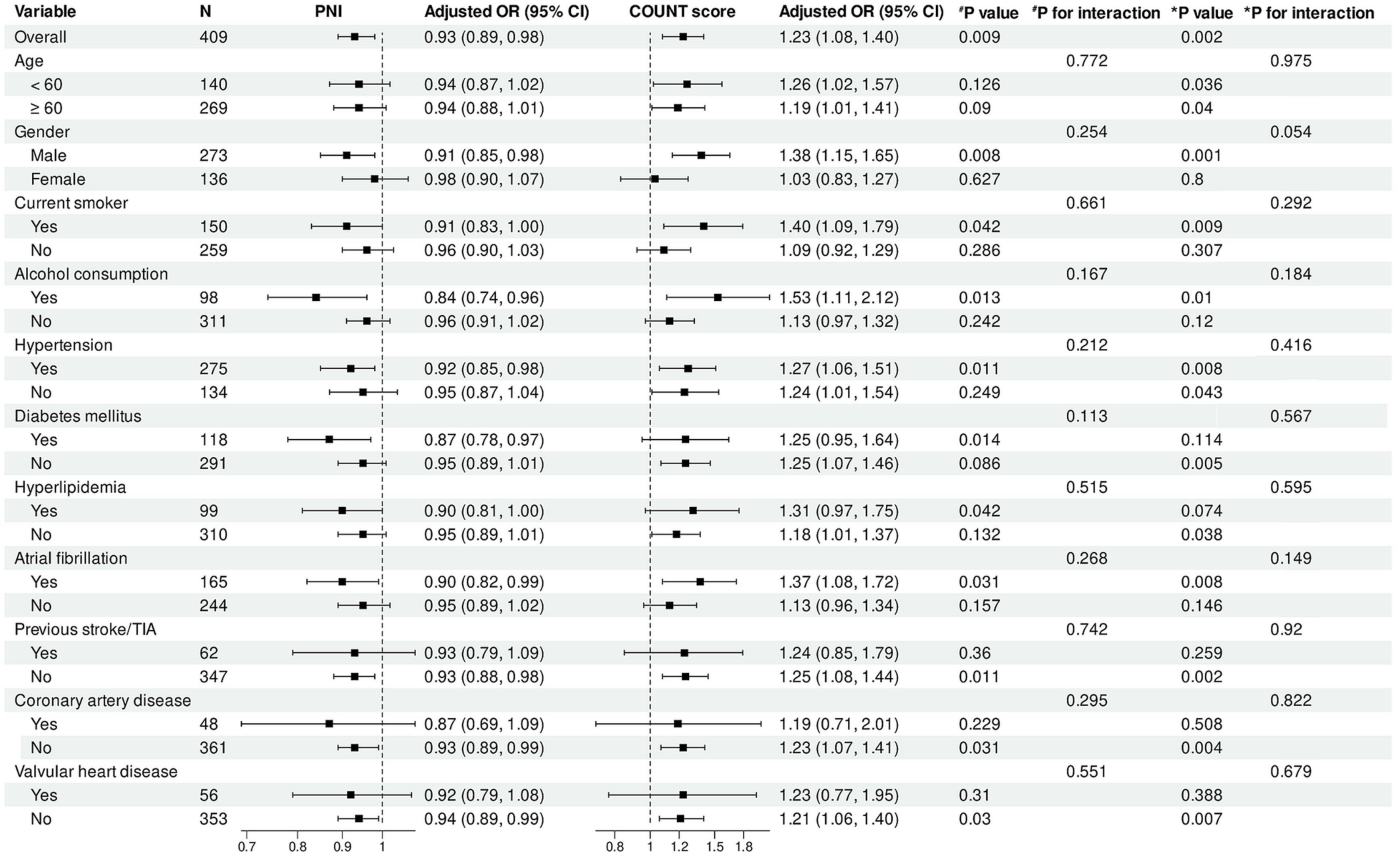
Subgroup analysis of association between PNI, CONUT score and functional outcomes. Forest plot showing subgroup analyses of the association between PNI (left panel) and CONUT score (right panel) with poor functional outcomes at 90 days. Odds ratios were adjusted for baseline NIHSS score, number of thrombectomy attempts, puncture-to-reperfusion time, white blood cell count, red blood cell count, platelet count, and AST. PNI, Prognostic Nutritional Index; CONUT, Controlling Nutritional Status; OR, odds ratio; CI, confidence interval; TIA, transient ischemic attack.

**Figure 6:**
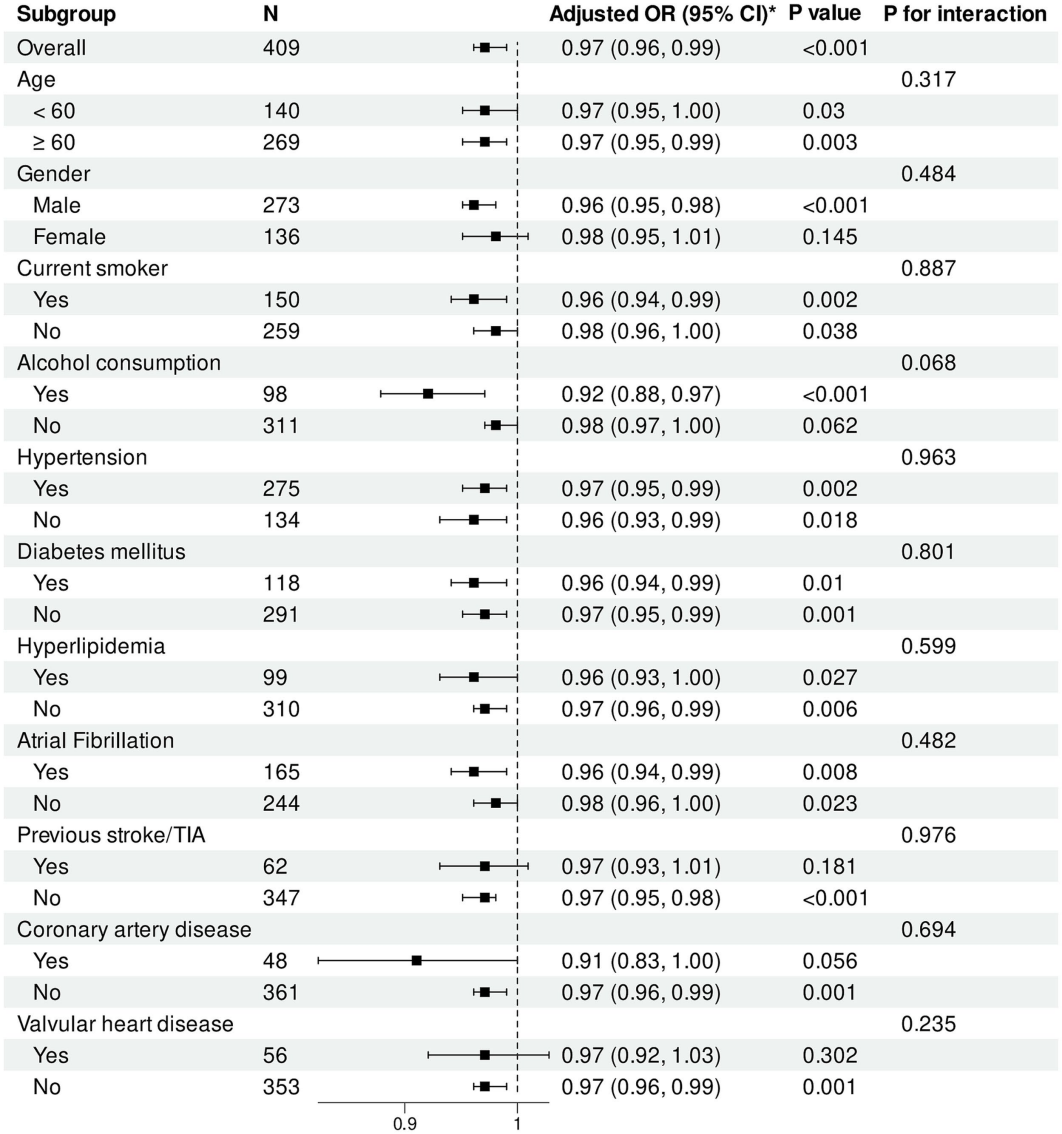
Subgroup analysis of association between HALP score and functional outcomes. Forest plot showing subgroup analysis of the association between HALP score and poor functional outcomes at 90 days. Odds ratios were adjusted for baseline NIHSS score, number of thrombectomy attempts, puncture-to-reperfusion time, white blood cell count, red blood cell count, platelet count, and AST. HALP, hemoglobin-albumin-lymphocyte-platelet; OR, odds ratio; CI, confidence interval; TIA, transient ischemic attack.

Subgroup analysis results (Figures 5, 6) demonstrated that in the overall cohort, each one-unit increase in PNI was significantly associated with reduced risk of poor functional outcomes (adjusted OR = 0.93, 95% CI: 0.89–0.98, *p* = 0.009). This protective association was more pronounced in specific populations, including male patients (*p* = 0.008), alcohol consumers (*p* = 0.013), and patients with comorbid hypertension (*p* = 0.011), diabetes (*p* = 0.014), or atrial fibrillation (*p* = 0.031). Additionally, statistically significant associations were observed in subgroups without history of previous stroke/TIA (*p* = 0.011) and without coronary artery disease (*p* = 0.031).

For CONUT score, in the overall population, each one-unit increase was significantly associated with increased risk of poor functional outcomes (adjusted OR = 1.23, 95% CI: 1.08–1.40, *p* = 0.002). This adverse association remained significant across multiple subgroups, including male patients (*p* = 0.001), patients aged <60 years (*p* = 0.036) and ≥60 years (*p* = 0.040), as well as current smokers (*p* = 0.009), alcohol consumers (*p* = 0.010), and patients with hypertension (*p* = 0.008) or atrial fibrillation (*p* = 0.008).

Regarding HALP score, in the overall population, each one-unit increase was significantly associated with reduced risk of poor functional outcomes (adjusted OR = 0.97, 95% CI: 0.96–0.99, *p* < 0.001). The protective effect of HALP score was particularly pronounced in male patients (*p* < 0.001). HALP score demonstrated significant protective associations in subgroups with and without hypertension, diabetes, hyperlipidemia, and atrial fibrillation (all *p* < 0.05).

Importantly, no statistically significant interactions were observed between the three nutritional indices and any subgroup variables (all interaction *p* values >0.05), indicating consistency in the impact of nutritional status on functional outcomes across different patient populations.

## 4 Discussion

In this single-center retrospective cohort study, we systematically evaluated the associations between three nutritional indices and 90-day functional outcomes in LVO-AIS patients who underwent EVT. Our investigation yielded several important insights. First, the prevalence of malnutrition risk in LVO-AIS patients varied substantially depending on the assessment tool used. PNI identified 15.9% of patients as having malnutrition risk (9.1% moderate, 6.8% severe), whereas the CONUT scoring system classified up to 81.9% of patients as having some degree of malnutrition risk (56.2% mild, 25.7% moderate-to-severe). Second, after adjusting for potential confounders, both PNI and HALP scores remained significantly associated with 90-day functional outcomes, with each one-unit increase corresponding to a 6 and 3% reduction in the risk of poor outcomes, respectively. Furthermore, our RCS analysis revealed a significant non-linear relationship between HALP score and functional outcomes, characterized by a steep risk reduction in the low-to-moderate value range, followed by a plateau effect. These associations maintained consistency across various demographic and clinical subgroups, underscoring the robustness of nutritional indices as prognostic markers.

Our findings align with existing evidence and further expand the understanding of the relationship between nutritional status and AIS outcomes. Growing evidence suggests that poor nutritional status serves as an independent predictor of various adverse clinical outcomes in AIS patients. Previous studies have confirmed that nutritional scores such as PNI and CONUT are significantly associated with early neurological deterioration, stroke-associated pneumonia, functional disability, mortality risk, and long-term stroke recurrence risk in AIS patients (8–14). Additionally, research evidence indicates that PNI and CONUT scores effectively predict complications related to acute reperfusion therapy in AIS. Studies have found that lower PNI and higher CONUT scores are independent risk factors for hemorrhagic transformation, all-cause mortality within 3 months, and poor functional outcomes at 3 months in AIS patients receiving intravenous thrombolysis (23–25). However, studies investigating the prognostic value of these nutritional indices in LVO-AIS patients undergoing EVT remain relatively limited. Özbek et al. (15) first reported that CONUT score was independently associated with in-hospital and long-term mortality after EVT (in-hospital mortality: OR = 1.426; 1-year all-cause mortality: OR = 1.2296; 3-year all-cause mortality: OR = 1.208, all *p* < 0.001). Subsequently, Luo et al. (16) confirmed that malnutrition risk assessed by CONUT score was independently associated with poor functional outcomes in LVO-AIS patients receiving EVT (OR = 1.387).

As a novel nutritional-inflammatory composite index, the HALP score has shown promise in predicting AIS-related complications in recent years. A limited number of studies have preliminarily confirmed associations between HALP score and various clinical outcomes in AIS, including stroke-associated pneumonia, hemorrhagic transformation, long-term mortality, and stroke recurrence risk (17–20). However, its prognostic predictive value in AIS patients receiving EVT has not been systematically validated. The present study not only confirmed the predictive value of CONUT score demonstrated in previous research but also systematically evaluated the application of PNI and HALP scores in EVT-treated patients for the first time. This provides new evidence to support clinical risk stratification and individualized treatment decision-making.

PNI and CONUT scores primarily assess nutritional status based on serum albumin, lymphocyte count, and total cholesterol levels. These three parameters may influence the prognosis of LVO-AIS patients undergoing EVT through multiple biological mechanisms. During reperfusion injury following AIS, albumin exerts neuroprotective effects through several mechanisms (26–28): First, as the most abundant antioxidant protein in plasma, albumin directly scavenges free radicals via its free thiol group (Cys34), mitigating cellular damage caused by oxidative stress. Second, albumin can bind and neutralize various proinflammatory factors, modulating cytokine storms and inhibiting neuroinflammatory cascade reactions. Third, albumin possesses antithrombotic properties that reduce blood viscosity, inhibit platelet aggregation, and improve microvascular perfusion. Finally, albumin stabilizes endothelial barrier function, maintains blood–brain barrier integrity, and reduces post-reperfusion vascular leakage and cerebral edema. Lymphocytes, as critical effector cells of cellular immunity, play complex bidirectional regulatory roles in inflammatory responses and tissue repair processes following cerebral ischemia and reperfusion (29). Different lymphocyte subpopulations exhibit distinctly different functional characteristics in cerebral ischemia. Studies have shown that CD4 + and CD8 + T cells can exacerbate brain tissue damage during the acute phase by releasing various neurotoxic factors (such as IFN-*γ*, TNF-*α*, and IL-17), whereas regulatory T cells (Tregs) primarily suppress neuroinflammation and promote neural repair through secretion of anti-inflammatory cytokines such as IL-10 and TGF-β.

The HALP score integrates hemoglobin, albumin, lymphocyte, and platelet parameters, providing a more comprehensive assessment of systemic status. Anemia exacerbates ischemic stroke injury through multiple mechanisms (30): first, reduced oxygen carriers under anemic conditions directly worsen hypoxia in the penumbral region; second, anemia can disrupt cerebrovascular autoregulation, leading to fluctuations in cerebral perfusion pressure and altered oxygen delivery dynamics; furthermore, the hyperdynamic circulatory state induced by anemia upregulates endothelial cell adhesion molecule expression, activating inflammatory cascade reactions and promoting thrombus formation; lastly, anemia-associated proinflammatory mediators upregulate inflammatory markers such as inducible nitric oxide synthase and CXC chemokine receptor 4, further amplifying inflammatory responses and collectively contributing to deterioration of stroke outcomes. Elevated platelet counts can similarly lead to poor prognosis in AIS patients through various mechanisms. Ischemia-triggered platelet activation initiates a series of cascade reactions, including upregulation of P-selectin expression, formation of platelet-leukocyte aggregates, and release of extracellular vesicles (31). These processes collaboratively mediate the “no-reflow phenomenon,” characterized by microvascular occlusion, endothelial swelling, and surrounding tissue edema (32). These factors may ultimately lead to poor patient outcomes.

Moreover, the four parameters of the HALP score not only function independently but potentially interact through synergistic mechanisms throughout the pathological process of AIS. During reperfusion, hemoglobin-mediated oxygen delivery and albumin’s free radical scavenging function establish complementary defensive mechanisms. Sufficient hemoglobin ensures adequate oxygen supply to the ischemic penumbra, while albumin efficiently eliminates peroxides and superoxide anion radicals through its Cys34 thiol residue, collectively mitigating oxidative stress-induced reperfusion injury (26, 30). Experimental research by Gekka et al. (33) demonstrated that integrating hemoglobin and albumin into a HemoAct complex (a hemoglobin core covalently bound to three albumin molecules) significantly enhances microvascular perfusion and tissue oxygenation efficiency, while effectively inhibiting cellular reactive oxygen species production under hypoxia/reoxygenation conditions, providing direct experimental evidence for their synergistic effect. Between lymphocytes and platelets, a complex immune-coagulation bidirectional regulatory network exists. Activated platelets interact with lymphocytes through surface integrin receptors, CD40-CD40L complexes, and P-selectin binding to PSGL-1 on lymphocyte surfaces, significantly enhancing activated T-cell adhesion to fibronectin. P-selectin preferentially binds to helper T lymphocytes (Th cells), thereby selectively promoting their tissue infiltration in inflammatory environments (34, 35). Furthermore, platelets simultaneously express MHC class I molecules and the co-stimulatory molecule CD86, directly presenting antigens to CD8+T cells and promoting their activation (36), consequently exacerbating ischemic neuronal injury.

The findings of our study have important clinical implications for both acute management and long-term prognosis improvement in LVO-AIS patients. Our RCS analysis revealed, for the first time, a significant non-linear relationship between HALP score and poor outcomes, characterized by a steep risk reduction as scores increased within the lower range, followed by a plateau effect. This phenomenon suggests that interventions targeting patients with moderate-to-severe malnutrition risk may yield more substantial clinical benefits. Based on this finding, we propose a stratified intervention strategy: for patients with severe malnutrition risk (PNI < 35 or CONUT≥5), prompt initiation of comprehensive nutritional assessment and intensive intervention protocols should be implemented, including early enteral nutritional support, intravenous albumin supplementation, and appropriate micronutrient supplementation. For long-term management, nutritional status assessment should become a routine component of stroke follow-up. Our data support integrating nutritional indices into existing prognostic assessment models; although their predictive capability is moderate when used independently, CONUT score demonstrated good sensitivity (0.70) and negative predictive value, making it suitable as a clinical risk screening tool. Notably, combining nutritional indices with established clinical-radiological scoring systems can significantly improve overall predictive accuracy, as confirmed in previous research (16). Moreover, recent studies have demonstrated that various composite indices based on peripheral blood markers, such as the stress hyperglycemia ratio (SHR), show significant value in predicting stroke outcomes. Large prospective cohort studies have confirmed that these composite biomarkers are closely associated with neurological functional recovery, disability severity, and long-term survival rates. Integrating these novel biomarkers into existing predictive models may substantially enhance their discriminative ability and predictive accuracy. Systematically incorporating nutritional assessment and intervention measures into acute management pathways may not only improve short-term neurological recovery but also has the potential to reduce long-term disability and recurrent events. However, prospective randomized controlled trials are needed to further validate the clinical benefits of these intervention strategies.

Several limitations of this study warrant consideration. First, as a single-center retrospective study, selection bias is inevitable and may limit the generalizability of our results. Although we employed progressively adjusted multivariable regression models to control for known confounders, this traditional approach may lead to model overfitting and might not select the most appropriate variable set. Adopting more principled causal inference methods, such as directed acyclic graph (DAG) analysis, might be more appropriate. DAG determines the minimal sufficient adjustment set based on established causal relationships, ensuring that selected variables are both necessary and sufficient to control for confounding factors while avoiding the introduction of unintended bias. Second, our study collected only single-point nutritional indicator data within 24 h of admission, which cannot reflect pre-stroke baseline levels or capture dynamic trends from the acute to recovery phases. Stress responses following AIS can trigger a series of metabolic changes, including decreased albumin levels, lymphocyte redistribution, and short-term fluctuations in total cholesterol; this “acute phase stress response” may affect the precise assessment of nutritional status. Additionally, the nutritional assessment indices used in this study were primarily based on serum biochemical markers, lacking comprehensive evaluation of body composition (such as muscle mass and fat distribution), functional parameters (such as grip strength and walking speed), and nutritional intake and absorption status. Despite these limitations, our study provides important evidence for nutritional status as a predictor of functional outcomes in LVO-AIS patients. Future research should employ prospective multicenter designs, integrate more comprehensive nutritional assessment methods, track dynamic changes in nutritional indicators from the acute to recovery phases, and explore the impact of individualized nutritional interventions on functional recovery.

## 5 Conclusion

This study confirms that nutritional status at admission is an independent predictor of 90-day functional outcomes in LVO-AIS patients undergoing EVT. Furthermore, our RCS analysis revealed a significant non-linear relationship between HALP score and functional outcomes. These findings emphasize the importance of incorporating nutritional assessment into standardized acute stroke management protocols, which can facilitate early identification of high-risk patients and optimize individualized treatment decisions.

## Data Availability

The datasets presented in this article are not readily available because the datasets used and/or analyzed during the current study are available from the corresponding author upon reasonable request. Requests to access the datasets should be directed to Renjing Zhu, zhurenjing@163.com.
