# Supplementary material for: Objective nutritional indices as an independent predictor of functional outcome after endovascular therapy for acute ischemic stroke: a cohort study in a Chinese population
Source: Front Nutr. 2025 Jun 18;12:1504208. doi: 10.3389/fnut.2025.1504208 (PMC12213872; doi:10.3389/fnut.2025.1504208)
Supplement: Supplementary file 1 [file Table_1.docx]

| **Supplementary Table 1.** Assessment of Multicollinearity Among Covariates Used in Different Nutritional Status Models for Predicting Functional Outcomes After Endovascular Therapy | | | | | | | |
| --- | --- | --- | --- | --- | --- | --- | --- |
| **Term** | VIF | VIF_CI_low | VIF_CI_high | SE_factor | Tolerance | Tolerance_CI_low | Tolerance_CI_high |
| **PNI** |  |  |  |  |  |  |  |
| PNI | 1.730266 | 1.53421 | 1.998276 | 1.315396 | 0.57794577 | 0.50043134 | 0.65180143 |
| Age | 1.417302 | 1.277523 | 1.627484 | 1.190505 | 0.70556584 | 0.61444536 | 0.78276491 |
| Current smoker | 1.12447 | 1.049061 | 1.315784 | 1.06041 | 0.8893082 | 0.76000326 | 0.95323341 |
| Hypertension | 1.132097 | 1.054357 | 1.321016 | 1.064 | 0.88331686 | 0.75699324 | 0.94844521 |
| Diabetes mellitus | 1.058527 | 1.00996 | 1.343905 | 1.028847 | 0.94470912 | 0.74410041 | 0.99013792 |
| Atrial Fibrillation | 1.387741 | 1.253529 | 1.593001 | 1.178024 | 0.72059551 | 0.6277459 | 0.79774765 |
| Baseline NIHSS score | 1.087549 | 1.0252 | 1.30416 | 1.042856 | 0.91949842 | 0.76677731 | 0.97541917 |
| PRT | 1.166922 | 1.079528 | 1.350353 | 1.080241 | 0.85695564 | 0.74054722 | 0.9263309 |
| NOTA | 1.107535 | 1.037692 | 1.306802 | 1.052395 | 0.90290573 | 0.76522671 | 0.96367752 |
| WBC | 37.377118 | 31.13908 | 44.906274 | 6.113683 | 0.02675434 | 0.0222686 | 0.03211399 |
| NEU | 36.582183 | 30.478782 | 43.949255 | 6.048321 | 0.02733571 | 0.02275351 | 0.03280971 |
| RBC | 1.460692 | 1.312862 | 1.678372 | 1.208591 | 0.68460709 | 0.5958154 | 0.76169459 |
| PLT | 1.166491 | 1.079209 | 1.349952 | 1.080042 | 0.85727182 | 0.74076712 | 0.92660467 |
| AST | 1.081225 | 1.021528 | 1.306461 | 1.03982 | 0.92487649 | 0.76542681 | 0.97892541 |
| **COUNT score** |  |  |  |  |  |  |  |
| COUNT score | 1.724308 | 1.529298 | 1.991167 | 1.313129 | 0.57994277 | 0.50221811 | 0.65389496 |
| PNI | 1.409091 | 1.27085 | 1.617888 | 1.187051 | 0.7096776 | 0.61808977 | 0.7868748 |
| Age | 1.142157 | 1.061478 | 1.328711 | 1.068717 | 0.87553673 | 0.75260894 | 0.94208273 |
| Current smoker | 1.131485 | 1.053929 | 1.320575 | 1.063713 | 0.88379404 | 0.75724567 | 0.94883028 |
| Hypertension | 1.059154 | 1.010238 | 1.341796 | 1.029152 | 0.9441496 | 0.74526976 | 0.98986601 |
| Diabetes mellitus | 1.386963 | 1.252899 | 1.592096 | 1.177694 | 0.72099964 | 0.62810268 | 0.79814897 |
| Atrial Fibrillation | 1.08599 | 1.02428 | 1.304539 | 1.042109 | 0.92081846 | 0.76655436 | 0.9762951 |
| Baseline NIHSS score | 1.168263 | 1.080523 | 1.351607 | 1.080862 | 0.85597155 | 0.73986008 | 0.92547767 |
| PRT | 1.111512 | 1.040307 | 1.308508 | 1.054283 | 0.89967515 | 0.76422925 | 0.96125481 |
| NOTA | 36.677622 | 30.558057 | 44.064154 | 6.056205 | 0.02726458 | 0.02269418 | 0.03272459 |
| WBC | 36.211947 | 30.171253 | 43.503529 | 6.017636 | 0.0276152 | 0.02298664 | 0.03314413 |
| NEU | 1.435313 | 1.292176 | 1.648572 | 1.198045 | 0.69671233 | 0.60658558 | 0.77388847 |
| RBC | 1.180794 | 1.089891 | 1.363625 | 1.086643 | 0.84688774 | 0.73333921 | 0.91752333 |
| PLT | 1.085535 | 1.024013 | 1.304671 | 1.04189 | 0.92120488 | 0.76647653 | 0.97654968 |
| **HALP score** |  |  |  |  |  |  |  |
| HALP.score | 3.567311 | 3.056801 | 4.204531 | 1.888733 | 0.28032323 | 0.23783864 | 0.32713938 |
| Age | 1.408492 | 1.270364 | 1.617189 | 1.186799 | 0.70997904 | 0.6183568 | 0.78717579 |
| Current smoker | 1.129416 | 1.052485 | 1.31911 | 1.06274 | 0.88541372 | 0.75808679 | 0.95013265 |
| Hypertension | 1.129755 | 1.052721 | 1.319348 | 1.062899 | 0.88514764 | 0.75795031 | 0.9499192 |
| Diabetes mellitus | 1.062861 | 1.011933 | 1.331127 | 1.030951 | 0.94085684 | 0.75124329 | 0.98820723 |
| Atrial Fibrillation | 1.384002 | 1.2505 | 1.588653 | 1.176436 | 0.72254206 | 0.62946396 | 0.79967987 |
| Baseline NIHSS score | 1.08803 | 1.025485 | 1.304065 | 1.043087 | 0.91909267 | 0.76683294 | 0.97514801 |
| PRT | 1.164488 | 1.077727 | 1.348096 | 1.079114 | 0.85874656 | 0.74178704 | 0.92787917 |
| Number of thrombectomy attempts | 1.105796 | 1.03656 | 1.306152 | 1.051568 | 0.90432588 | 0.76560765 | 0.96472983 |
| WBC | 78.899387 | 65.628813 | 94.894878 | 8.882533 | 0.01267437 | 0.01053798 | 0.01523721 |
| NEU | 76.713754 | 63.813354 | 92.263595 | 8.758639 | 0.01303547 | 0.01083851 | 0.0156707 |
| RBC | 1.34916 | 1.222345 | 1.548304 | 1.161533 | 0.74120217 | 0.64586818 | 0.81809982 |
| PLT | 2.073666 | 1.81799 | 2.409257 | 1.440023 | 0.48223784 | 0.41506573 | 0.55005811 |
| AST | 1.087023 | 1.024888 | 1.304275 | 1.042604 | 0.91994402 | 0.76670932 | 0.97571593 |
| **PNI grade** |  |  |  |  |  |  |  |
| PNI grade | 1.324766 | 1.202967 | 1.519655 | 1.150985 | 0.75485037 | 0.6580441 | 0.83127793 |
| Age | 1.404409 | 1.267331 | 1.611777 | 1.185078 | 0.71204311 | 0.62043328 | 0.78905984 |
| Current smoker | 1.127927 | 1.05157 | 1.317346 | 1.062039 | 0.88658189 | 0.75910192 | 0.9509594 |
| Hypertension | 1.141794 | 1.06135 | 1.327719 | 1.068548 | 0.87581485 | 0.75317124 | 0.9421965 |
| Diabetes mellitus | 1.064367 | 1.012701 | 1.326198 | 1.031681 | 0.93952596 | 0.75403521 | 0.98745826 |
| Atrial Fibrillation | 1.399138 | 1.263051 | 1.605631 | 1.182852 | 0.71472559 | 0.62280826 | 0.79173398 |
| Baseline NIHSS score | 1.089357 | 1.02636 | 1.30291 | 1.043723 | 0.91797243 | 0.76751262 | 0.97431694 |
| PRT | 1.172025 | 1.083476 | 1.354505 | 1.082601 | 0.85322379 | 0.73827702 | 0.92295516 |
| Number of thrombectomy attempts | 1.111847 | 1.040633 | 1.307868 | 1.054441 | 0.89940447 | 0.76460314 | 0.96095324 |
| WBC | 30.886059 | 25.759294 | 37.074394 | 5.557523 | 0.03237707 | 0.02697279 | 0.03882094 |
| NEU | 30.384294 | 25.342316 | 36.470606 | 5.512195 | 0.03291174 | 0.02741934 | 0.03945969 |
| RBC | 1.37907 | 1.246777 | 1.582284 | 1.174338 | 0.72512616 | 0.63199762 | 0.80206812 |
| PLT | 1.150923 | 1.067929 | 1.335316 | 1.072811 | 0.86886767 | 0.74888641 | 0.93639145 |
| AST | 1.087522 | 1.025264 | 1.303203 | 1.042843 | 0.91952163 | 0.7673403 | 0.97535858 |
| **HALP score group** |  |  |  |  |  |  |  |
| HALP score group | 3.726071 | 3.191041 | 4.39175 | 1.930303 | 0.26837922 | 0.22769967 | 0.31337739 |
| Age | 1.435878 | 1.29323 | 1.64792 | 1.198281 | 0.69643796 | 0.60682564 | 0.7732575 |
| Current smoker | 1.137582 | 1.058478 | 1.323693 | 1.066575 | 0.87905745 | 0.75546202 | 0.94475304 |
| Hypertension | 1.141166 | 1.061032 | 1.326516 | 1.068254 | 0.87629646 | 0.75385447 | 0.94247863 |
| Diabetes mellitus | 1.06164 | 1.011463 | 1.331463 | 1.030359 | 0.94193865 | 0.75105347 | 0.98866703 |
| Atrial Fibrillation | 1.394004 | 1.259161 | 1.599008 | 1.18068 | 0.71735781 | 0.62538791 | 0.79417948 |
| Baseline NIHSS score | 1.088599 | 1.025987 | 1.302065 | 1.04336 | 0.91861154 | 0.768011 | 0.97467089 |
| PRT | 1.17372 | 1.084897 | 1.355472 | 1.083383 | 0.85199211 | 0.73775057 | 0.92174629 |
| Number of thrombectomy attempts | 1.101589 | 1.034043 | 1.303151 | 1.049566 | 0.90777986 | 0.76737094 | 0.96707748 |
| WBC | 73.447272 | 61.157282 | 88.248078 | 8.570138 | 0.01361521 | 0.01133169 | 0.01635128 |
| NEU | 70.48977 | 58.698363 | 84.690904 | 8.395819 | 0.01418646 | 0.01180764 | 0.01703625 |
| RBC | 1.394411 | 1.259492 | 1.599481 | 1.180852 | 0.71714844 | 0.62520266 | 0.79397114 |
| PLT | 1.974038 | 1.736609 | 2.287998 | 1.405005 | 0.5065758 | 0.43706329 | 0.57583501 |
| AST | 1.083134 | 1.02277 | 1.303525 | 1.040737 | 0.92324647 | 0.76715032 | 0.97773677 |
| **COUNT grade** |  |  |  |  |  |  |  |
| COUNT.grade2 | 1.707125 | 1.51555 | 1.969888 | 1.30657 | 0.58578017 | 0.50764317 | 0.65982635 |
| Age | 1.408865 | 1.270952 | 1.616977 | 1.186956 | 0.70979099 | 0.61843793 | 0.78681202 |
| Current smoker | 1.152055 | 1.068752 | 1.336292 | 1.073338 | 0.86801445 | 0.7483396 | 0.93567112 |
| Hypertension | 1.134962 | 1.056496 | 1.322407 | 1.065346 | 0.88108699 | 0.75619673 | 0.94652526 |
| Diabetes mellitus | 1.063247 | 1.012166 | 1.328788 | 1.031138 | 0.94051563 | 0.7525656 | 0.98797996 |
| Atrial Fibrillation | 1.380586 | 1.248005 | 1.584045 | 1.174984 | 0.72432998 | 0.63129531 | 0.80127899 |
| Baseline NIHSS score | 1.088703 | 1.025968 | 1.302998 | 1.043409 | 0.91852446 | 0.76746076 | 0.97468958 |
| PRT | 1.174185 | 1.08509 | 1.356569 | 1.083598 | 0.85165479 | 0.73715387 | 0.92158289 |
| Number of thrombectomy attempts | 1.110523 | 1.039756 | 1.307254 | 1.053814 | 0.90047661 | 0.76496213 | 0.9617637 |
| WBC | 36.603332 | 30.510476 | 43.954144 | 6.050069 | 0.02731992 | 0.02275098 | 0.03277563 |
| NEU | 35.512182 | 29.603706 | 42.641133 | 5.95921 | 0.02815935 | 0.02345153 | 0.03377955 |
| RBC | 1.361125 | 1.232259 | 1.561489 | 1.166672 | 0.73468658 | 0.64041424 | 0.81151765 |
| PLT | 1.168954 | 1.081188 | 1.351598 | 1.081182 | 0.85546541 | 0.73986507 | 0.92490841 |
| AST | 1.083102 | 1.022676 | 1.304546 | 1.040722 | 0.92327419 | 0.76655022 | 0.97782669 |
| Multicollinearity assessment of variables used in multivariate models for different nutritional indices. The table shows variance inflation factors (VIF) and tolerance values with their corresponding 95% confidence intervals. Variables were categorized by multicollinearity severity using the following VIF thresholds: VIF<5 (low collinearity), 5≤VIF<10 (moderate collinearity), VIF≥10 (severe collinearity). Note the high VIFs for WBC and NEU (>30), indicating severe multicollinearity, with a strong correlation between them (r=0.98). Additionally, lymphocyte count was excluded from all models due to significant correlations (r>0.5) with PNI, CONUT, and HALP scores, as it is a component of these indices. Based on this analysis, only WBC was retained in the final multivariate models to avoid collinearity issues. Abbreviations: VIF, variance inflation factor; CI, confidence interval; SE, standard error; PNI, Prognostic Nutritional Index; CONUT, Controlling Nutritional Status; HALP, Hemoglobin-Albumin-Lymphocyte-Platelet; WBC, white blood cell count; NEU, neutrophil count; RBC, red blood cell count; PLT, platelet count; AST, aspartate aminotransferase; NIHSS, National Institutes of Health Stroke Scale; PRT, puncture-to-reperfusion time; NOTA, number of thrombectomy attempts. | | | | | | | |
