# Supplementary material for: Objective nutritional indices as an independent predictor of functional outcome after endovascular therapy for acute ischemic stroke: a cohort study in a Chinese population
Source: Front Nutr. 2025 Jun 18;12:1504208. doi: 10.3389/fnut.2025.1504208 (PMC12213872; doi:10.3389/fnut.2025.1504208)
Supplement: Supplementary file 2 [file Table_2.docx]

| **Supplementary Table 3.** Akaike Information Criterion Values for Restricted Cubic Spline Models With Different Numbers of Knots for Nutritional Assessment Scores | | | | | | | |
| --- | --- | --- | --- | --- | --- | --- | --- |
| **Nutritional Score and Model** | 3 Knots | 4 Knots | 5 Knots | 6 Knots | | 7 Knots | |
| **PNI** |  |  |  |  | |  | |
| Model 1 | 525.74 | 527.62 | 529.49 | | 531.08 | | 532.77 |
| Model 2 | 487.43 | 488.78 | 490.69 | | 491.87 | | 493.59 |
| Model 3 | 463.83 | 465.51 | 467.08 | | 467.87 | | 469.56 |
| **HALP socre** |  |  |  | |  | |  |
| Model 1 | 518.28 | 517.45 | 517.32 | | 518.55 | | 518.53 |
| Model 2 | 480.65 | 481.38 | 480.88 | | 482.17 | | 482.23 |
| Model 3 | 451.58 | 451.81 | 451.24 | | 452.36 | | 452.19 |
| Values represent Akaike Information Criterion (AIC) for restricted cubic spline models testing the non-linear association between nutritional scores and poor functional outcome (modified Rankin Scale score 3-6 at 90 days). Lower AIC values indicate better model fit. Model 1: Unadjusted. Model 2: Adjusted for demographic and clinical factors (age, Current smoker, hypertension, diabetes mellitus, atrial fibrillation, and baseline NIHSS score). Model 3: Additionally adjusted for procedural parameters and laboratory indices (number of thrombectomy attempts, puncture-to-reperfusion time, leukocytes, erythrocytes, platelet, and aspartate aminotransferase). Bold values indicate the optimal number of knots selected for each nutritional score in the corresponding model. Abbreviations: PNI, prognostic nutritional index; HALP, hemoglobin, albumin, lymphocyte, and platelet; NIHSS, National Institutes of Health Stroke Scale. | | | | | | | |
