# Supplementary material for: Objective nutritional indices as an independent predictor of functional outcome after endovascular therapy for acute ischemic stroke: a cohort study in a Chinese population
Source: Front Nutr. 2025 Jun 18;12:1504208. doi: 10.3389/fnut.2025.1504208 (PMC12213872; doi:10.3389/fnut.2025.1504208)
Supplement: Supplementary file 3 [file Table_3.docx]

| **Supplementary Table 2.** Performance Metrics of Logistic Regression Models for Nutritional Assessment Scores in Predicting Poor Functional Outcome After Endovascular Therapy | | | | | | | | | | | |
| --- | --- | --- | --- | --- | --- | --- | --- | --- | --- | --- | --- |
| **Name** | **Model** | **AIC**  **(weights)** | **AICc**  **(weights)** | **BIC**  **(weights)** | **Tjur's R2** | **RMSE** | **Sigma** | **Log_loss** | **Score_log** | **Score_spherical** | **PCP** |
| **PNI** | | | | | |  |  |  |  |  |  |
| Model 1 | glm | 524.0(<.001) | 524.0(<.001) | 532.0(<.001) | 0.036 | 0.472 | 1.130 | 0.636 | -270.660 | 0.003 | 0.555 |
| Model 2 | glm | 485.9(<.001) | 486.3(<.001) | 518.0(<.001) | 0.157 | 0.440 | 1.083 | 0.574 | -Inf | 0.002 | 0.611 |
| Model 3 | glm | 462.2(>.999) | 463.3(>.999) | 518.4(>.999) | 0.232 | 0.419 | 1.048 | 0.531 | -Inf | 0.003 | 0.645 |
| **Nutritional status by PNI** | | | | | | | | | | | |
| Model 1 | glm | 533.0(<.001) | 533.1(<.001) | 545.1(<.001) | 0.018 | 0.476 | 1.139 | 0.644 | -268.071 | 0.036 | 0.547 |
| Model 2 | glm | 488.9(<.001) | 489.4(<.001) | 525.0(0.564) | 0.153 | 0.441 | 1.085 | 0.576 | -Inf | 0.002 | 0.609 |
| Model 3 | glm | 465.3(>.999) | 466.6(>.999) | 525.5(0.436) | 0.228 | 0.421 | 1.051 | 0.532 | -Inf | 0.002 | 0.644 |
| **COUNT score (continuous)** | | | | | |  |  |  |  |  |  |
| Model 1 | glm | 516.5(<.001) | 516.5(<.001) | 524.5(0.003) | 0.055 | 0.467 | 1.122 | 0.626 | -276.527 | 0.008 | 0.563 |
| Model 2 | glm | 481.1(<.001) | 481.4(<.001) | 513.2(0.849) | 0.169 | 0.436 | 1.077 | 0.569 | -Inf | 0.002 | 0.616 |
| Model 3 | glm | 460.5(>.999) | 460.5(>.999) | 516.7(0.148) | 0.237 | 0.418 | 1.046 | 0.529 | -Inf | 0.003 | 0.648 |
| **Nutritional status by PNI** | | | | | | | | | | | |
| Model 1 | glm | 520.5(<.001) | 520.6(<.001) | 532.6(0.001) | 0.053 | 0.468 | 1.126 | 0.629 | -270.958 | 0.014 | 0.563 |
| Model 2 | glm | 484.1(<.001) | 484.6(<.001) | 520.2(0.603) | 0.168 | 0.437 | 1.079 | 0.570 | -Inf | 0.003 | 0.616 |
| Model 3 | glm | 460.9(>.999) | 462.1(>.999) | 521.1(0.396) | 0.243 | 0.416 | 1.046 | 0.527 | -Inf | 0.003 | 0.651 |
| **HALP score** | | | | | | | | | | | |
| Model 1 | glm | 526.7(<.001) | 526.7(<.001) | 534.7(<.001) | 0.032 | 0.473 | 1.133 | 0.639 | -267.331 | 0.002 | 0.553 |
| Model 2 | glm | 483.8(<.001) | 484.1(<.001) | 515.9(0.180) | 0.163 | 0.438 | 1.080 | 0.572 | -Inf | 0.002 | 0.614 |
| Model 3 | glm | 456.6(>.999) | 57.7(>.999) | 512.8(0.820) | 0.246 | 0.415 | 1.042 | 0.524 | -Inf | 0.003 | 0.652 |
| **HALP quartiles** | | | | | |  |  |  |  |  |  |
| Model 1 | glm | 516.0(<.001) | 516.1(<.001) | 532.0(<.001) | 0.067 | 0.464 | 1.120 | 0.621 | -276.279 | 0.017 | 0.569 |
| Model 2 | glm | 479.7(<.001) | 480.2(<.001) | 519.8(0.055) | 0.182 | 0.433 | 1.073 | 0.562 | -Inf | 0.003 | 0.622 |
| Model 3 | glm | 449.9(>.999) | 451.3(>.999) | 514.1(0.945) | 0.270 | 0.408 | 1.031 | 0.511 | -Inf | 0.002 | 0.663 |
| This table presents comprehensive performance metrics for logistic regression models evaluating the predictive ability of different nutritional assessment scores for poor functional outcome (modified Rankin Scale score 3-6 at 90 days) after endovascular therapy. Model 1: Unadjusted. Model 2: Adjusted for demographic and clinical factors (age, smoking status, hypertension, diabetes mellitus, atrial fibrillation, and baseline NIHSS score). Model 3: Additionally adjusted for procedural parameters and laboratory indices (number of thrombectomy attempts, puncture-to-reperfusion time, white blood cell count, red blood cell count, platelet count, and aspartate aminotransferase).  Performance metrics are categorized as: (1) Goodness-of-fit indices (AIC, AICc, and BIC with model weights in parentheses); (2) Discrimination ability (Tjur's R²); (3) Prediction accuracy measures (RMSE and Sigma); (4) Calibration metrics (Log_loss and Score_spherical); and (5) Classification performance (PCP, proportion of correct predictions). Lower values of AIC, AICc, BIC, RMSE, Sigma, and Log_loss indicate better model performance, while higher values of Tjur's R², Score_spherical, and PCP indicate improved performance. The "-Inf" values observed in the Score_log metric for Models 2 and 3 indicate potential perfect prediction for some observations, which occurs when the model assigns probabilities very close to 0 or 1 for certain covariate patterns. Multiple performance metrics were used to evaluate model quality and assess potential overfitting. The consistent improvement in goodness-of-fit indices (decreasing AIC, AICc, and BIC values), coupled with concurrent enhancement in discrimination (increasing Tjur's R²), accuracy (decreasing RMSE and Sigma), calibration (decreasing Log_loss), and classification metrics (increasing PCP) across models of increasing complexity, suggests the absence of overfitting. The high model weights (approaching 1) for Model 3 across all nutritional scores indicate strong evidence that the fully adjusted models provide the best fit without sacrificing parsimony. Additionally, the similar patterns of improvement observed across different nutritional assessment tools and between continuous and categorical representations of the same scores further supports the robustness of these associations.  Abbreviations: AIC, Akaike information criterion; AICc, corrected Akaike information criterion; BIC, Bayesian information criterion; RMSE, root mean square error; PCP, proportion of correct predictions; PNI, prognostic nutritional index; CONUT, controlling nutritional status; HALP, hemoglobin, albumin, lymphocyte, and platelet; NIHSS, National Institutes of Health Stroke Scale. | | | | | | | | | | | |
