# Supplementary material for: Objective nutritional indices as an independent predictor of functional outcome after endovascular therapy for acute ischemic stroke: a cohort study in a Chinese population
Source: Front Nutr. 2025 Jun 18;12:1504208. doi: 10.3389/fnut.2025.1504208 (PMC12213872; doi:10.3389/fnut.2025.1504208)
Supplement: Supplementary file 4 [file Image_1.pdf]

**Supplementary Figure 1.** Patient Selection Flowchart

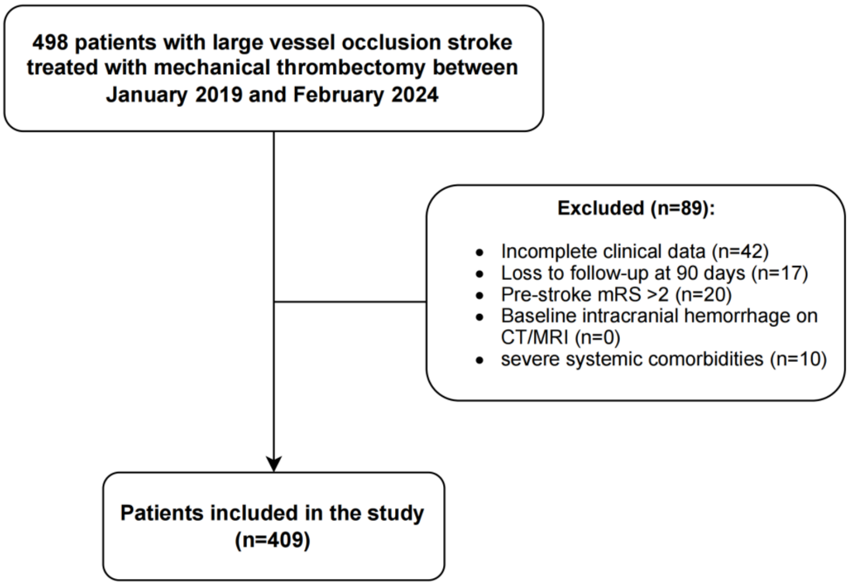


Flow diagram illustrating the patient selection process. Among 498 consecutive patients with large vessel occlusion acute ischemic stroke who underwent endovascular therapy between January 2019 and December 2024, 89 patients were excluded based on predefined criteria. The most common exclusion reason was incomplete clinical data (n=42), primarily due to missing hematological or biochemical parameters. Other exclusions included: loss to follow-up at 90 days (n=17), pre-stroke modified Rankin Scale score >2 (n=20), baseline intracranial hemorrhage on CT/MRI (n=0), and severe systemic comorbidities (n=10). The final analysis included 409 patients who met all inclusion criteria and had complete 90-day functional outcome data. Abbreviations: LVO-AIS, large vessel occlusion acute ischemic stroke; EVT, endovascular therapy; CT, computed tomography; MRI, magnetic resonance imaging; mRS, modified Rankin Scale.
