# Supplementary material for: Objective nutritional indices as an independent predictor of functional outcome after endovascular therapy for acute ischemic stroke: a cohort study in a Chinese population
Source: Front Nutr. 2025 Jun 18;12:1504208. doi: 10.3389/fnut.2025.1504208 (PMC12213872; doi:10.3389/fnut.2025.1504208)
Supplement: Supplementary file 5 [file Image_2.pdf]

Supplementary Figure 2. Pairwise Comparison of ROC Curves for Nutritional Indices in Predicting Poor Functional Outcomes

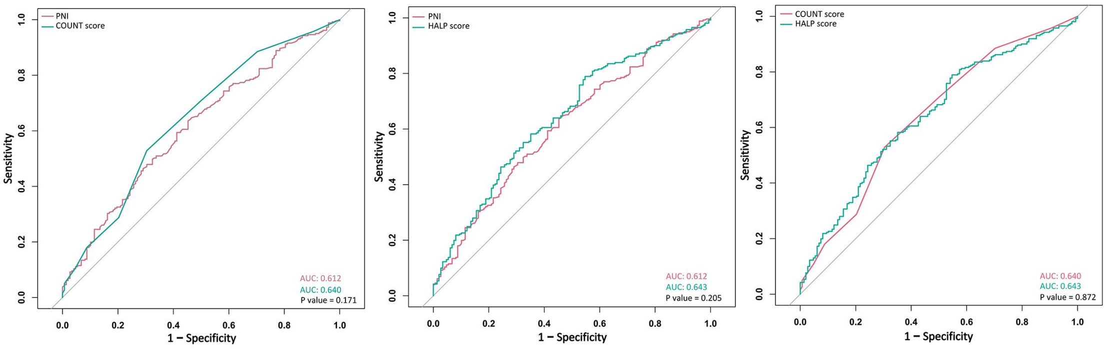

Pairwise comparison of receiver operating characteristic (ROC) curves for the three nutritional indices in predicting poor functional outcomes (mRS 3-6) at 90 days after endovascular therapy for large vessel occlusion acute ischemic stroke. Left panel: comparison between PNI (red, AUC: 0.612) and CONUT score (blue, AUC: 0.640), P=0.171. Middle panel: comparison between PNI (red, AUC: 0.612) and HALP score (blue, AUC: 0.643), P=0.205. Right panel: comparison between CONUT score (red, AUC: 0.640) and HALP score (blue, AUC: 0.643), P=0.872. DeLong tests were used to assess statistical significance of differences between AUCs. Although CONUT and HALP scores demonstrated numerically higher discriminative performance compared to PNI, no statistically significant differences were observed between any pair of nutritional indices (all P>0.05). The diagonal gray line represents an AUC of 0.5 (no discriminative ability). Abbreviations: ROC, receiver operating characteristic; AUC, area under the curve; PNI, Prognostic Nutritional Index; CONUT, Controlling Nutritional Status; HALP, Hemoglobin-Albumin-Lymphocyte-Platelet; mRS, modified Rankin Scale.
